# Low-Dose Chemotherapy with Insulin (Insulin Potentiation Therapy) in Combination with Hormone Therapy for Treatment of Castration-Resistant Prostate Cancer

**DOI:** 10.5402/2012/140182

**Published:** 2012-05-08

**Authors:** Christo Damyanov, Desislava Gerasimova, Ivan Maslev, Veselin Gavrilov

**Affiliations:** Medical Center “Integrative Medicine”, Deliiska Vodenitza Street, Bl. 330, 1592 Sofia, Bulgaria

## Abstract

*Purpose*. To evaluate the results and quality of life of patients with resistant of castration-resistant tumors previously treated with Insulin-potentiation therapy (IPT) combined with hormone therapy. *Materials and methods*. Sixteen patients with metastasis prostate tumors after bilateral castration, androgenic blockade, and progression of the disease were observed during the study. The patients were divided into two groups: group A consisting of 8 patients treated with low-dose chemotherapy Epirubicin, Vinblastine, and Cyclophosphamide combined with LHRH agonist and group B consisting of another 8 patients treated with low-dose chemotherapy Docetaxel combined with LHRH agonist. *Results*. The overall (groups A and B) results concerning PSA after the sixth IPT show partial effect in 8 out of 16 (50%) patients, stabilization in 4 out of 16 (25%), and progression in 4 out of 16 (25%). The median survival for all treated patients is 11,7 months (range 3–30 months). During the treatment no significant side effects were observed, and no lethal cases occurred. *Conclusion*. In spite of the small number of the treated patients with castration-resistant prostate tumors, the preliminary results are promising and this gives us hope and expectations for future serious multicenter research over the possibilities for routine implementation of IPTLD.

## 1. Introduction

In spite of the efficacy of standard androgen deprivation therapy for metastasis tumors of prostate gland, almost all of the patients progress with their disease. In the last few years the prostate tumors progress although the androgen blockade is defined as castration-resistant prostate cancer—CRPC [1, 2].

Despite obvious efforts for revealing the reasons for hormonal resistance after androgen deprivation, the treatment remains a challenge.

 The duration of remission after treatment of hormone-resistant tumors of prostate gland with secondary hormonal manipulation is short and there is no serious impact on survival [3].

Up to 1990 the results after chemotherapy for hormone-resistant tumors of prostate gland are disappointing. Later a Canadian researcher drew our attention to the potentialities of the combination Mitoxantrone with Prednisone where an improvement in pain and quality of life was indicated but there was no effect on the survival [3, 4].

In 2004 two trials registered and prolonged survival after using Docetaxel. The overall survival with Docetaxel was 18,9 months against 16,5 months with Mitoxantrone and Prednisolone. There were registered improvements also in toxicity and quality of life. At this moment the treatment with Docetaxel in combination with Prednisone is accepted as standard of care for metastatic castration-resistant prostate tumors [5, 6].

In a process of investigation there are some new chemotherapeutic agents like Epothilones and Satraplatin [7, 8].

In standard chemotherapy used for treatment of diverse tumors, we usually use the maximum tolerated dose (MDT) as we intend to achieve better results. Despite many clinical researches with different combinations of chemotherapeutics, the progress in effectiveness is very modest and there are many toxic effects.

On the other hand prolonged intervals between the applications of the chemotherapeutics are factors which contribute to chemoresistance.

In searching other possibilities for reducing the toxic effects of chemotherapy without decreasing its antitumor efficacy in the last few years intensive researches have been made on chemotherapy in low doses and in short intervals between the applications—the so-called metronomic chemotherapy. Preclinical research and small clinical research show serious possibilities for lowering the toxic effects without diminishing the efficacy [9, 10].

 Usage of low doses chemotherapeutics with increased frequency suggests another method called insulin-potentiated therapy (IPT) or now called insulin-potentiated targeted low-dose therapy (IPTLD), where standard schemes of chemotherapy are used in combination with intravenous insulin, 10 times lower doses of chemotherapeutics, and short intervals between the applications. This treatment has very low toxicity and our personal experience shows that efficiency is not deferring from the standard chemotherapy [11].

 According to our previous experience in implementation of IPTLD in different tumors including castration resistant prostate, tumors we conducted a research for new possibilities where our main target was to improve the quality of life [12, 13].

This study is focusing on the potential of IPTLD in combination with hormone therapy for treatment of patients with castration-resistant prostate tumors.

## 2. Patients and Method

 Between April 2006 and May 2011 a total of 406 patients with diverse tumors were treated with IPTLD and 21 out of them were with prostate cancer. Sixteen of them were with advanced prostate cancer (Stage III-Stage IV and nodal, bone, or visceral secondary) and cynically apparent hormonal independence entered the study.

 They were divided into two groups: group A: 8 patients treated with Epirubicin, Vinblastine, and Cyclophosphamide in combination with LHRH agonist (Goserelin depot 3,6 mg). In group B another 8 patients were treated with Docetaxel in combination with LHRH agonist (Goserelin depot 3,6 mg).

Before the treatment all patients gave informed content. The main requirement for eligibility was objective and subjective data for progression of the disease after surgical castration, androgenic therapy, and androgen withdrawal. In Table 1 the clinical characteristics of treated patients are presented. 

Pretreatment evaluation of the patients includes history of the disease, physical exam, Karnofsky performance status (KPS), subjective condition evaluated with Beretta [14], FBC, biochemistry including AF, prostate-specific antigen (PSA), urine analysis, chest radiographs, ultrasound, bone scan, and CT.

Control lab exams include tumor markers after 6th IPT application and after every fourth IPT after that and control exams of bone scan and CT—after the 10th application and after that on 3- or 6-month intervals from the beginning. Every month patients complete Berettas questionnaire for their subjective state and we note only sections A and B in Table 4.

## 3. Treatment

### 3.1. Insulin Potentiation Therapy (IPT)

1. Group A: insulin i.v. (0,4 UI/kg.) in combination with Cyclophosphamide (0,10–0,15 g/m^2^)/Epirubicin (3 mg/m^2^); Vinblastine (0,5 mg/m^2^) i.v. in 8 patients.
2. Group B: insulin i.v. (0,4 UI/kg.) in combination with Docetaxel (3,6 mg/m^2^) i.v. in 8 patients.

Length of one scan treatment: 6 applications in every 5-day interval, then sustaining treatment in gradual increasing intervals (four applications in 10 days, 2, 3, and more weeks).

In the interval, have Dexamethason, 20 mg; Cyclophosphamide, 50 mg p.o.; Doxycyclin, 100 mg; Legalon, 3 × 140 mg; Celebrex, 2 × 7,5 mg; antioxidants, and ozone therapy.

Maintaining treatment consists of no more than 24 IPT applications.

**Hormone Therapy**

Six application (one per month) with LHRH agonist (Goserelin depot 3, 6 mg) in every 28 days for six months.

**Objective Response**

to treatment was assessed as per PSA levels and bone scan results. A complete response required normal PSA levels and normal bone scan. A partial response required a decrease of >50% of the baseline value of PSA and decrease in the measurable lesions seen on the bone scan and also the absence of signs of disease progression. Stabilization of disease was defined as decrease of <50% of the baseline levels of PSA, an absence of increase of lesions seen on the bone scan. Patients were considered to have disease progression if they showed an increase in 2 successive PSA level measurements and/or appearance of new neoplastic lesions.

**Additional Parameters**

For evaluation the effect of the treatment includes mean remission duration and median overall survival.

**Quality of Life**

It was reported every month with Beretta Questionnaire.

**The Toxicity**

The toxicity of treatment is recorded according to the criteria of World Health Organization (WHO).

## 4. Results

 The mean follow-up period was 7,6 months (2 to 23 months). Sixteen patients finished the core cycle of 6 applications. After 6th IPT application 3 patients of group A and 4 patients of group B discontinued the treatment because of social reasons. In group A we implemented 159 IPT applications and in group B 106.

 Treatment is very well tolerated by the patients. During the treatment no significant side effects were observed, and no lethal cases occurred. Common side effects include light weakness and sleepiness on the day of the procedure.

 Two (16) patients complained of nausea and vomiting a few hours after the procedure but this disappears on the next day. Slight reduce in hemoglobin is observed in 6 of 16 patients. Blood transfusion was necessary before treatment in 3 and during it in 2 patients with low starting hemoglobin. Slight increase in liver enzymes is observed in 5 from 16. These lab changes did not influence the subjective status of the patients. Slight increase in creatinine and urea is observed in one patient after previous nephrectomy. Changes in lab results are in parameters of grade 0 of WHO criteria.

 The results after 6th IPT concerning PSA criteria for both groups show partial effect in 8 of 16 p. (50%), stabilization in 4 out of 16 (25%), and progression in 4 out of 16 (25%). Usually the improvement occurs after the first 2 IPT applications. The results after 10th IPT show complete response in 3 of 9 (33%), partial response in 1 of 9 (11%), stabilization in 2 of 9 (22%) and progression in 3 of 9 (33%).

 In Table 2 are presented the results of treatment according to PSA criteria after the end of the core 6 weekly course of IPT. In Table 3 are presented the results after 10th IPT or 3 months after the beginning.

 The mean values of PSA in those who responded to the treatment decrease from 256,7 to 94,6 ng/mL in group A and from 2215,3 ng/mL to 956,2 ng/mL in group B after 6th (course of treatment) IPT. After the 10th course of treatment the values were, respectively, 36,3 nag/mL in group A and 4,9 nag/mL in group B.

 The mean duration of the remission in group A is 7,6 months (range 3–18 months) and for group B it is10 months (range 3–18 months). The median survival for all treated patients is 11,7 months (range 3–30 months).

Subjective improvement is observed in all patients in both groups, average from 24,2 points before treatment to 8,9 points after 6th IPTLD. More than 50% decrease in symptomatic index is observed in 7 (8) from group A and 3 (8) from group B.

**Figure 1:**
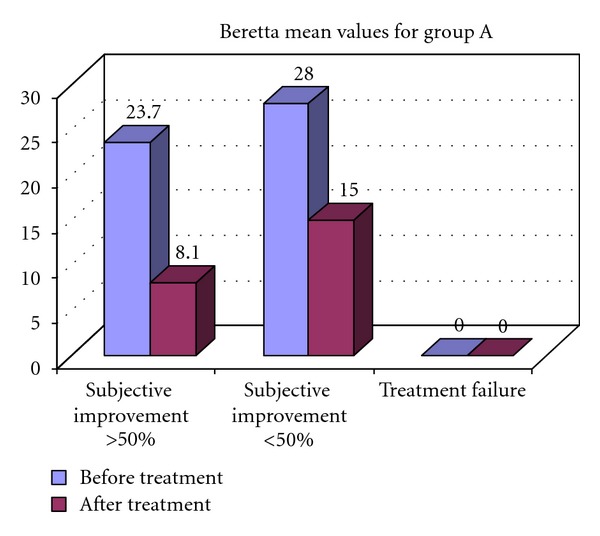
Quality of life assessment according to Berreta Symptoms Index, after the 6th IPT course for group A.

**Figure 2:**
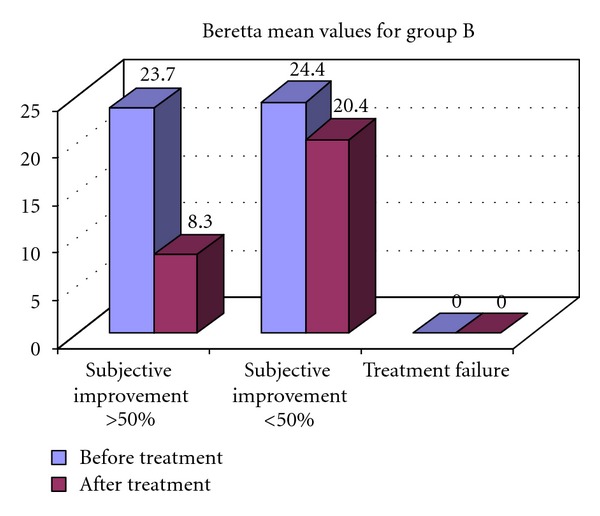
Quality of life assessment according to Berreta Symptoms Index, after the 6th IPT course for group B.

 In Figures 1 and 2 are presented the results from the subjective improvement in both groups after 6th IPT.

## 5. Discussion

 The method of insulin potentiation therapy was empirically invented in 1930 from Mexican doctor D. Perez Garsia, who was applying it successfully for the treatment of chronic and oncology diseases for 41 years. Lately his practice is continued by his son and grandson who now gathers more and more popularity and the method is used in practice from increasing number of doctors (more then 400) and clinics all around the world. 

 The theoretical conception for the mechanisms of action of IPT is explained in two publications of Ayre S. G., D. Perez Garcia y Bellon, and D. Perez Garcia, in 1986 and 2000 [10, 16]. Same authors in 1990 submitted in European Journal of Cancer one case from their practice demonstrating complete tumor regression of ductal breast carcinoma in 32-year woman explaining the mechanisms and method that they had use [17].

 In 2003 Lasalvia-Prisco et al. published in Cancer Chemotherapy and Pharmacology the first clinical research investigating the effect of the combination of insulin and Methotrexate in patients with breast cancer [18].

Basic role for the efficacy of the method plays the usage of hormone insulin in diabetics. Despite of the diversity of the actions of the insulin in the human body not all of them are unrevealed. In 1960s many investigations were conducted and they show that besides its effect in lowering the blood sugar insulin has serious effect in the whole metabolism:

- increases the permeability of cell membrane,
- influences the metabolic processes in human body with the increase of the regenerating processes,
- facilitates the transport of intra and extra cellular liquids which helps the organism to eliminate the toxic products,
- have other endocrine effects: directly stimulates suprarenal gland to produce epinephrine and glucocorticoid hormones and stimulates ACTH secretion. These endocrine effects also have a positive influence on the regenerating processes.

Various researches show that insulin hormone has a significant impact also on the tumor cells themselves. As a result of the current knowledge of the effect of insulin on biology of tumor cells several conclusions can be done.

- Increased permeability after the insulin effect on the cellular membrane results in increased intracellular quantity of antitumor agents.
- Insulin influences the intracellular metabolism of the tumor cell, which leads to increase of the number of cells in phase S, where they are with highly sensitive to specific chemotherapeutics.
- The increased number of insulin receptors on the tumor cell, in comparison to the normal one, allows the before mentioned 2 factors to act predominantly.

Despite the serious achievements in the field of revealing the intimate mechanisms of the action of the insulin hormone in the human body we are still far away from getting answers to all our questions. Future researches will probably reveal more details regarding the effective clinical usage of insulin.

 Having in mind the serious problems of the treatment of hormone-resistant prostate tumors and taking into consideration previous experimental researches, we conducted a clinical research on the direct inhibitory effect of LHRH analog Triptorelin acetate depot on the cellular proliferation. The results confirmed local inhibitory action from the experimental researches and showed that they can successfully be combined with chemotherapy [19, 20].

In search of a new method for lowering the toxicity of the chemotherapy for treating oncological diseases, we began in 2006 to implement IPTLD combined with LHRH agonist Gosrelin depot 3,6 mg. Among the all treated patients 21 are with advanced prostate tumors, 16 are hormone-resistant, and those 16 are divided according to the used chemotherapeutics into 2 groups—group A and group B.

 After the first 6 IPT applications overall (groups A and B) response to treatment on PSA criteria shows partial effect and stabilization in 12 of 16 (75%) patients. After the 10th IPTLD application or 3 months after starting treatment, complete response, partial response, and stabilization were observed in 4 of 9 (66.6%), while in 3 of 9 (33.3%) was registered complete effect.

 Symptomatic improvement of the treatment after the sixth IPTLD is observed in all patients in both groups. More than 50% improvement in symptomatic index was reported in 10 of 16 (62.5%) for the same period.

 The mean duration of remission in patients with complete response in both groups was 17 (range 15–18 months) months. In one patient treated with Docetaxel, after a 15-month remission that progressed we used again IPTLD with Cyclophosphamide, Epirubicin, and Vinblastin in combination with I LHRH agonist. After the sixth application the PSA values decreased from 399,9 ng/mL to 35,0 ng/mL.

 Despite the advanced stage of disease in patients treated by us, the treatment is well tolerated without any serious side effects. Quality of life after the second IPTLD application is significantly improved, and this applies even to patients with treatment failure in terms of PSA criteria.

The reasons for discontinuation of the treatment are mainly social and primarily financial. The research was carried out in a private medical centre and the financial expenses are not covered by the National Health Insurance Institution. This reflects the limited opportunities for long-term monitoring of health outcomes. Still the small number of treated patients and the short-term of the follow-up do not allow us to do a serious comparative analysis of the results of the treatment in both groups, as well as making definitive conclusions regarding the effectiveness of the treatment. The preliminary presented results give us reason to assume that extensive comparative researches are necessary for better proving the effectiveness of the method.

## 6. Conclusion

Our present experience with IPTLD (in more than 400 treated patients) with various tumors as well as the practical experience of the growing number of doctors practicing the method gives us a reason to assume that IPTLD method provides a real opportunity for resolving one of the most serious problems of toxicity associated with chemotherapy using maximum tolerated doses. A certain advantage of the method along with its effectiveness is the significantly improved quality of life of the treated patients.

In spite of the small number of patients treated by us with castrate-resistant prostate tumor, the preliminary results are promising and this gives us hope and expectations for future serious researches on the potential of widespread clinical use of IPTLD.

## Figures and Tables

**Table 1:** Clinical characteristics of the treated patients.

Clinical parameters	Group A	Group B
Total number of patients	8	8

Age		

Median	64	66
Range	56–76	53–73

Karnovsky performance scale (mean)		

Median	68	74
Range	50–80	60–90

Beretta score before treatment		

Median	24	24
Range	10–42	6–33

Glison score		

4	1	0
5	1	1
6	0	1
7	2	1
8	1	0
9	3	3
Unknown	0	2

Tumor stage		

Stage III	0	0
Stage IV	8	8

Extend of disease (nr.)		

With local metastases	3 (8)	3 (8)
With distant metastases	7 (8)	7 ( 8)

Previous therapy		

Surgical orchiectomy	6 (8)	5 (8)
Antiandrogens	8 (8)	7 (8)
Palliative radiotherapy	4 (8)	0

PSA(ng/mL)		

Median	389,6	1511,2
Range	63,3–1320	17,16–8395

Al. phosphatase mg/mL		

Median	1673	1120
Range	246–4286	253–5264

**Table 2:** Treatment results (after 6 IPT).

	Group A	Group B	Overall
	*n*	*n*	*n *(%)
Complete response	0	0	0
Partial response	5 (8)	3 (8)	8/16 (50)
Stabile disease	2 (8)	2 (8)	4/16 (25)
Progressive disease	1 (8)	3 (8)	4/16 (25)

**Table 3:** Treatment results (after 10 IPT).

	Group A	Group B	Overall
	*n*	*n*	*n* (%)
Complete response	1 (5)	2 (4)	3/9 (33)
Partial response	0 (5)	1 (4)	1/9 (11)
Stabile disease	2 (5)	0 (4)	2/9 (22)
Progressive disease	2 (5)	1 (4)	3/9 (33)

**Table 4:** Beretta Self-Compilation Questionnaire.

Self-Compilation Questionnaire for weekly determination of subjective status					

The patient should cross the circle corresponding to the worst feeling in the week					

Subjective factors	Coding definition				
	0	1	2	3	4
	No	Little	Enough	Much	Very Much
*Section A*					

(1) Feeling ill	○	○	○	○	○
(2) Feeling bad	○	○	○	○	○
(3) Feeling anxious	○	○	○	○	○
(4) Feeling depressed	○	○	○	○	○
(5) Presence of nausea	○	○	○	○	○
(6) Losing appetite	○	○	○	○	○
(7) Reduced working capacity (usual job)	○	○	○	○	○
(8) Reduced housework and concentration capacity	○	○	○	○	○
(9) Reduced social activities	○	○	○	○	○
(10) Reduced sexual activity	○	○	○	○	○

*Section B*					

(11) Presence of fatigue	○	○	○	○	○
(12) Presence of respiratory distress	○	○	○	○	○
(13) Presence of pain	○	○	○	○	○

*Section C*					

(14) Is treatment helping?	○	○	○	○	○
(15) Is doctor helping?	○	○	○	○	○
(16) Is nursing staff helping?	○	○	○	○	○
(17) Is hospital centre helping?	○	○	○	○	○
(18) Is family helping?	○	○	○	○	○
(19) Is social “milieu” helping?	○	○	○	○	○
(20) Is any other person/service/institution helping?	○	○	○	○	○

See [15].
